# Prehabilitation in Cardiovascular Surgery: Concepts, Evidence, and Future Direction

**DOI:** 10.7759/cureus.106795

**Published:** 2026-04-10

**Authors:** Abubakar I. Sidik, Malik K Al-Ariki, Hasan Saghir, Nawid A Rahimi, Mohamad K Sleiman, Maxim Tur, Anvar K Djumanov, Alina V Ogurchikova, Ivan Karpenko, Vladislav Dontsov, Gazinur R Galiev, Grigorii Esion

**Affiliations:** 1 Department of Cardiovascular Surgery, Peoples' Friendship University of Russia, Moscow, RUS; 2 Department of Hospital Surgery and Pediatric Surgery, Peoples' Friendship University of Russia, Moscow, RUS; 3 Department of Medicine, Novosibirsk State Medical University, Novosibirsk, RUS; 4 Department of Cardiovascular Disease, N.I. Pirogov Russian National Research Medical University, Moscow, RUS; 5 Department of Cardiovascular Medicine, Volgograd State Medical University, Volgograd, RUS; 6 Department of Cardiovascular Medicine, Novosibirsk State Medical University, Novosibirsk, RUS; 7 Department of Surgical Diseases No. 2, Tashkent Medical University, Tashkent, UZB; 8 Department of Cardiovascular Medicine, Peoples' Friendship University of Russia, Moscow, RUS; 9 Department of Cardiothoracic Surgery, A.A. Vishnevskiy Hospital, Moscow, RUS; 10 Department of Cardiothoracic Surgery, Moscow Regional Research and Clinical Institute, Moscow, RUS; 11 Department of Cardiovascular Medicine, First Moscow State Medical University Named After I.M. Sechenov, Moscow, RUS; 12 Department of Cardiovascular Surgery, A.A. Vishnevskiy Hospital, Moscow, RUS

**Keywords:** adult malnutrition, cardiovascular and thoracic surgery, exercise training, functional capacity, inspiratory muscle training, perioperative optimisation, physical frailty, prehabilitation, surgical outcomes research, sarcopenia

## Abstract

Patients undergoing cardiovascular surgery are increasingly older and multimorbid, with a high prevalence of frailty, sarcopenia, malnutrition, and psychological vulnerability, all of which are strongly associated with adverse postoperative outcomes yet remain incompletely captured by conventional risk models. Prehabilitation has emerged as a proactive, multimodal strategy to address these modifiable vulnerabilities in the preoperative period. This narrative review synthesizes contemporary evidence on prehabilitation in adult cardiovascular surgery, focusing on its core components, reported clinical benefits, feasibility, and barriers to implementation. Current evidence indicates that exercise-based prehabilitation, particularly programs incorporating aerobic training and inspiratory muscle training, is safe and feasible and improves preoperative functional capacity, with consistent reductions in postoperative pulmonary complications and hospital length of stay. Nutritional optimization, smoking and alcohol cessation, psychological preparation, and targeted comorbidity management further support physiological resilience and patient readiness for surgery, although these domains remain underrepresented in cardiac-specific trials. Despite growing interest, the evidence base is limited by heterogeneous program designs, variable outcome measures, short intervention windows, and a lack of adequately powered randomized trials. Integration of prehabilitation into established perioperative care pathways, improved patient selection using frailty and fitness assessment, and scalable delivery models, including tele-prehabilitation, are needed to support wider adoption. Overall, prehabilitation represents a promising patient-centered strategy to improve recovery and resilience in adult cardiovascular surgery.

## Introduction and background

Cardiovascular surgery is increasingly offered to older, multimorbid patients who carry a substantial burden of modifiable vulnerability factors such as frailty, malnutrition, and sarcopenia. Large cardiovascular cohorts and expert reviews now recognize sarcopenia and related muscle wasting syndromes as independent cardiovascular risk factors that interact with traditional comorbidities to worsen outcomes in this population [1]. In patients scheduled for coronary artery or valvular procedures, a recent proportional meta-analysis of more than 600,000 individuals reported pooled frailty and pre-frailty prevalences of 28% and 40%, respectively, underscoring how common these syndromes are among candidates for cardiovascular surgery [2].

Frailty itself is strongly prognostic. A systematic review and meta-analysis of 66,448 cardiac surgical patients found that both frailty and pre-frailty were associated with significantly higher operative mortality, perioperative complications, and non-home discharge compared with robust patients [3]. Contemporary narrative and quantitative syntheses confirm that frail cardiovascular surgery patients have approximately double the risk of operative mortality, longer intensive care and hospital stays, higher rates of postoperative delirium, and worse mid-term survival, even after adjustment for conventional risk scores [3,4]. These data suggest that standard risk models alone are insufficient to capture the vulnerability of the modern cardiac surgical cohort and highlight the need for strategies that modify frailty-related risk before surgery.

Malnutrition and sarcopenia are closely intertwined with frailty in this setting and represent particularly attractive preoperative targets. In an adult cardiovascular surgery cohort, nutritional indices used to screen for sarcopenia correlated with worse postoperative outcomes, supporting the concept that poor nutritional reserves and muscle depletion confer additive risk beyond traditional factors [5]. A dedicated systematic review and meta-analysis showed that sarcopenia in cardiovascular surgery is associated with increased early and late mortality and poorer functional recovery, independent of age and comorbidity burden [6]. Together with broader cardiovascular data linking sarcopenia to disability, hospitalization, and death [1], this evidence positions malnutrition and loss of muscle mass/strength as central, but potentially modifiable, determinants of surgical resilience.

Prehabilitation has emerged as a multimodal strategy to address exactly these domains of vulnerability. Typically delivered in the weeks prior to surgery, cardiac prehabilitation programs combine structured exercise (aerobic, resistance, and sometimes inspiratory muscle training), nutritional optimization, psychological support, and targeted management of comorbidities such as anemia, diabetes, and chronic lung disease [7,8]. By improving cardiorespiratory fitness, muscle strength, nutritional status, and coping capacity, prehabilitation seeks to enhance patients’ physiological reserve so they can better tolerate the inflammatory, metabolic, and functional stress of major cardiac operations and recover more rapidly. Conceptually, this shifts the focus from merely “risk stratifying” frail, sarcopenic, or malnourished patients to actively modifying their risk profile in the preoperative window.

The empirical evidence base for prehabilitation in cardiovascular surgery, while growing, remains heterogeneous and partly conflicting. An early systematic review of preparative rehabilitation reported that preoperative exercise interventions are generally safe and feasible and may improve preoperative functional capacity and some perioperative outcomes, but highlighted small sample sizes, diverse protocols, and variable endpoints [9]. More focused work on inspiratory muscle training (IMT) has shown that short, intensive preoperative IMT can reduce postoperative pulmonary complications and shorten postoperative hospital stay [10], and a meta-analysis of preoperative IMT in cardiovascular surgery patients confirmed a significant reduction in length of stay, although effects on mechanical ventilation time and ICU stay were inconsistent [11].

More recently, an exercise-based prehabilitation meta-analysis concluded that programs combining aerobic and resistance training before cardiovascular surgery are effective and safe, with improvements in six-minute walk distance and signals toward fewer postoperative complications in selected subgroups [12,13]. Narrative overviews of cardiac prehabilitation emphasize wide variation in program components, timing, and intensity, and the use of disparate outcome measures across trials, which complicates evidence synthesis and guideline development [7].

While several recent systematic reviews and meta-analyses have evaluated specific components of prehabilitation in cardiovascular surgery, particularly exercise training and IMT, these studies are typically limited to single interventions or narrowly defined outcome measures. As a result, they do not fully capture the multidimensional and clinically integrated nature of prehabilitation as it is applied in contemporary practice. This narrative review aims to complement rather than duplicate existing syntheses by providing a comprehensive, multimodal perspective that integrates exercise, nutritional optimization, psychological preparation, behavioral risk modification, and comorbidity management within a unified framework. In addition, it contextualizes the evidence within real-world clinical pathways, addressing implementation challenges, feasibility, and models of delivery, and highlights gaps specific to cardiovascular surgical populations. By synthesizing these domains into a single clinically oriented framework, this review seeks to bridge the gap between fragmented evidence and practical application in cardiovascular surgical care.

## Review

Search strategy (narrative approach)

This narrative review applied a deliberately broad literature search to identify sources related to prehabilitation in adult cardiovascular surgery. Electronic searches were performed using five major databases: PubMed, Embase, Scopus, the Cochrane Library, and Google Scholar. The search process was flexible rather than protocol-based, allowing progressive refinement of search terms as themes became clearer during screening.A wide range of free-text and controlled vocabulary terms was used. Core keywords included “prehabilitation,” “preoperative exercise,” “cardiovascular surgery,” “cardiovascular surgery,” “nutrition,” “frailty,” “rehab,” and “multimodal therapy.” Variations of these terms, such as “preoperative rehabilitation,” “exercise training before surgery,” “preoperative inspiratory muscle training,” and “nutrition optimization,” were also applied to increase search breadth and ensure inclusivity across different research traditions and terminology styles.

The primary timeframe spanned the last 20-25 years, reflecting the period during which prehabilitation has become a recognized perioperative concept in cardiovascular surgery. Earlier landmark papers were consulted selectively when cited in modern evidence to provide historical context regarding the development of frailty screening, exercise physiology in surgical patients, and nutritional strategies.

This review included a wide range of study formats, such as randomized controlled trials, observational cohorts, feasibility and pilot studies, systematic reviews, meta-analyses, expert consensus documents, and relevant clinical guidelines. Priority was given to studies reporting functional capacity, postoperative complications, mortality, length of stay, and feasibility or adherence measurements.

To maintain clinical relevance, the search excluded literature focused on pediatric cardiovascular surgery, congenital cardiac interventions, and non-surgical cardiac disease such as heart failure cohorts. Studies examining only postoperative rehabilitation without a preoperative intervention were also excluded.

This narrative search strategy was designed to favor comprehensiveness rather than methodological restriction. It supports the goals of this review by capturing the breadth of available knowledge, defining evidence gaps, and informing future directions for research and clinical practice in cardiac surgical prehabilitation.

Results

The initial database search yielded several hundred records, from which approximately 150 articles were reviewed in full text based on relevance to cardiovascular surgical prehabilitation. Additional references were identified through citation tracking of relevant reviews and landmark studies.

Types of Cardiovascular Surgery Addressed

Cardiac prehabilitation research applies to a broad spectrum of operative procedures. Although these surgeries share similar physiological stresses, their patient risk profiles differ according to age distribution, disease burden, urgency, and underlying cardiac dysfunction. Understanding these distinctions is central to evaluating how prehabilitation may be tailored to specific operative pathways.

Coronary artery bypass grafting (CABG): CABG remains among the most frequently performed major cardiac procedures worldwide and is often offered to older individuals with multiple comorbidities [14]. Patients face elevated preoperative risk due to advanced atherosclerotic disease, diabetes, chronic kidney disease, and reduced functional capacity [15]. Frailty prevalence in CABG candidates has been reported at more than 20%, contributing to prolonged recovery and poorer postoperative outcomes [3]. These factors make CABG a strong target for exercise-based and nutritional prehabilitation, as improving cardiorespiratory capacity and metabolic reserve may reduce complication rates and enhance functional recovery.

Major valve surgery: Valve surgery, particularly for degenerative mitral and aortic valve disease, involves a population that frequently presents at an advanced age with reduced physiological reserve and high frailty burden [16]. Patients undergoing valve repair or replacement often have symptoms of heart failure, sarcopenia, and malnutrition, which increase susceptibility to postoperative complications. Transcatheter and surgical aortic valve replacement populations demonstrate significant functional limitation preoperatively, and structured rehabilitation programs have shown benefit in improving physical performance and recovery, highlighting the relevance of preoperative conditioning strategies [17].

Aortic surgery: Open and endovascular procedures for thoracic and thoracoabdominal aortic aneurysm repair carry distinctive physiological challenges. These operations frequently require prolonged cardiopulmonary bypass, hypothermic circulatory arrest, and extensive reconstruction, which increase the risk of neurological injury, pulmonary dysfunction, renal impairment, and prolonged hospitalization [18]. Individuals presenting for aortic surgery also tend to have high frailty risk, respiratory compromise, and steep age-related decline in mobility. Prehabilitation interventions, particularly those aimed at inspiratory muscle function and aerobic capacity, may therefore be especially valuable for this cohort.

Left ventricular assist device (LVAD) implantation and cardiac transplantation: Prehabilitation interest is increasing among advanced heart failure patients referred for LVAD implantation or heart transplantation. These individuals frequently exhibit profound frailty, cachexia, and severe functional limitation, resulting in risk profiles far more extreme than those associated with CABG or isolated valve disease. Frailty has been independently associated with poorer survival in heart-transplant candidates, providing a strong rationale for interventions that improve physiological resilience prior to surgery [19]. Although cardiac-specific prehabilitation evidence remains limited in this subgroup, emerging data suggest that structured preoperative conditioning may improve eligibility, postoperative recovery, and long-term outcomes.

**Figure 1:**
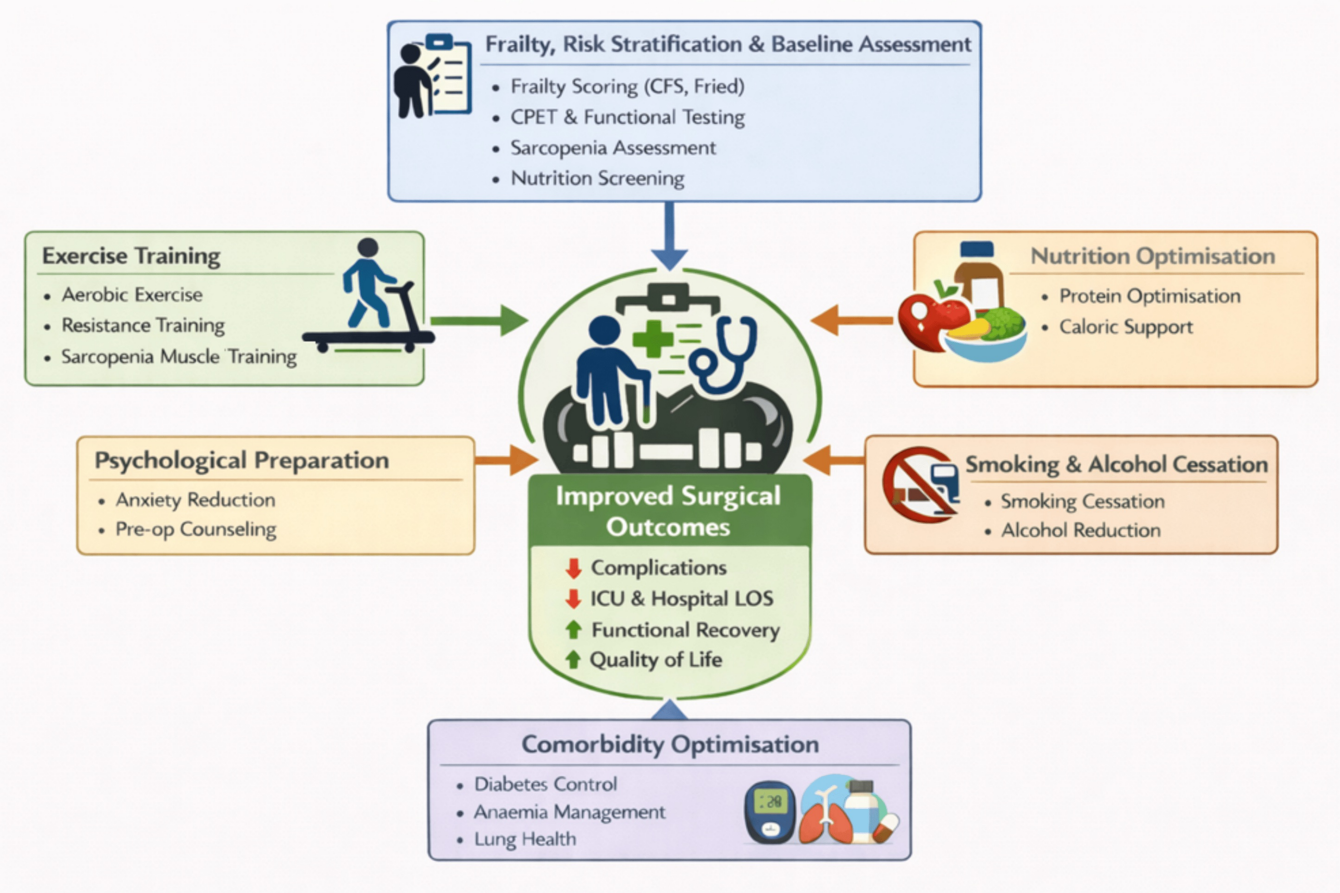
Multimodal prehabilitation framework in cardiovascular surgery Patients awaiting cardiovascular surgery undergo baseline assessment of frailty, functional capacity, sarcopenia, and nutritional status to identify modifiable vulnerabilities. Targeted multimodal interventions, including exercise training, nutritional optimization, smoking and alcohol cessation, psychological preparation, and comorbidity management, are implemented in the preoperative period to enhance physiological reserve and surgical readiness, ultimately improving postoperative recovery and reducing complications. This figure was created with the help of BioRender.

Figure 1 provides an integrated visual summary of the multimodal prehabilitation framework, illustrating how baseline frailty and risk assessment inform the key intervention domains addressed throughout this review.

Frailty, Risk Stratification, and Baseline Assessment

Prehabilitation in cardiovascular surgery depends on a thorough baseline assessment to identify high-risk patients who may benefit from intervention before the operation. Key domains include frailty, cardiorespiratory fitness, muscle mass and strength, and nutritional status. These factors influence postoperative outcomes and help tailor prehabilitation strategies. Table 1 summarizes commonly used tools for the assessment of frailty, malnutrition, and sarcopenia in patients undergoing cardiovascular surgery, highlighting the domains assessed, resource requirements, scoring systems, and key strengths and limitations of each instrument.

**Table 1:** Frailty, malnutrition, and sarcopenia assessment tools

References	Tool name	Domain(s) assessed	Required resources	Cut-offs/scoring	Pros	Limitations
Rockwood et al., 2005 [20]	Clinical Frailty Scale (CFS)	Global frailty, function	Clinical interview	Scale 1–9; ≥5 frail	Quick, validated, prognostic	Subjective
Fried et al., 2001 [21]	Fried frailty phenotype	Physical frailty	Grip dynamometer, stopwatch	≥3 criteria frail	Objective, validated	Time-consuming
Rolfson et al., 2006 [22]	Edmonton Frail Scale (EFS)	Multidimensional frailty	Questionnaire, clock test	≥8 frail	Includes cognition	Less cardiac-specific
Studenski et al., 2011 [23]	Gait speed	Physical performance	Stopwatch, walkway	<0.8 m/s	Simple, prognostic	Affected by comorbidity
Malmstrom and Morley, 2013 [24]	Hand-grip strength	Muscle strength	Hand dynamometer	<27 kg men, <16 kg women	Objective, fast	No muscle mass info
Su et al., 2019 [25]	CT-derived muscle area	Muscle mass	CT scan, software	Sex-specific cut-offs	Highly objective	Imaging required
Malmstrom and Morley, 2013 [24]	SARC-F questionnaire	Sarcopenia risk	Questionnaire	≥4 suggests sarcopenia	Very simple	Low sensitivity
Vellas et al., 1999 [26]	Mini Nutritional Assessment	Nutrition status	Questionnaire	<17 malnourished	Validated in the elderly	Less surgical-specific
Stratton et al., 2004 [27]	Malnutrition Universal Screening Tool (MUST)	Malnutrition risk	BMI, weight loss history	≥2 high risk	Widely used	Limited functional info
Kondrup et al., 2003 [28]	Nutritional Risk Screening 2002 (NRS-2002)	Nutrition risk	Questionnaire	≥3 at risk	Validated in surgery	Needs training
Detsky et al., 1987 [29]	Subjective Global Assessment (SGA)	Nutrition status	Clinical exam	A–C categories	Prognostic	Subjective

Frailty scoring: Frailty reflects diminished physiological reserve and is a strong predictor of adverse outcomes after cardiovascular surgery. Commonly used frailty instruments include the Clinical Frailty Scale (CFS) and the Fried frailty phenotype score. The CFS evaluates functional independence and comorbidity burden on a scale from very fit to severely frail and has been shown to correlate with increased mortality, complications, and length of hospital stay in surgical patients [3,20]. The Fried phenotype assesses five physical criteria, including weight loss, exhaustion, physical activity, walk speed, and grip strength, to categorize patients as robust, pre-frail, or frail, with frail patients facing substantially higher perioperative risk [21]. These frailty evaluations are increasingly incorporated into cardiac surgical risk assessment to identify patients who may derive greater benefit from prehabilitation.

Functional capacity testing and cardiopulmonary exercise testing (CPET): Objective assessment of aerobic capacity is critical in prehabilitation planning. CPET measures peak oxygen uptake (VO_2_ max) and anaerobic threshold, providing objective data on cardiovascular and pulmonary reserve. Reduced VO_2_ max is consistently linked to worse postoperative outcomes, including prolonged intensive care stay and increased morbidity [30]. Classic studies have demonstrated that lower CPET measures in the elderly and cardiac populations precede higher perioperative risk, supporting the use of VO_2_ max and related metrics to quantify baseline fitness and guide individualized exercise prescriptions within prehabilitation programs [31].

Sarcopenia assessment: Sarcopenia, defined as loss of muscle mass and strength, is an independent risk factor for poor outcomes in cardiovascular surgery. Assessment approaches include grip strength, gait speed, and imaging modalities such as computed tomography to quantify muscle area. A systematic review in cardiovascular surgery patients has linked sarcopenia with increased postoperative mortality, longer hospital stays, and poorer functional recovery, highlighting the importance of muscle assessment in risk stratification [6].

Nutritional screening: Malnutrition is common in patients undergoing cardiovascular surgery and is associated with increased postoperative complications, prolonged length of stay, and mortality. Preoperative nutritional screening aims to identify patients at risk using validated tools such as the Mini Nutritional Assessment (MNA), Malnutrition Universal Screening Tool (MUST), Nutritional Risk Screening 2002 (NRS-2002), and Subjective Global Assessment (SGA) [32]. Studies indicate that screening tools can predict adverse outcomes and help trigger early nutrition support and optimization strategies [33]. Given that malnutrition may be under-recognized using simple metrics like body mass index alone, structured screening is recommended in prehabilitation pathways to allow timely dietary intervention and optimization.

**Figure 2:**
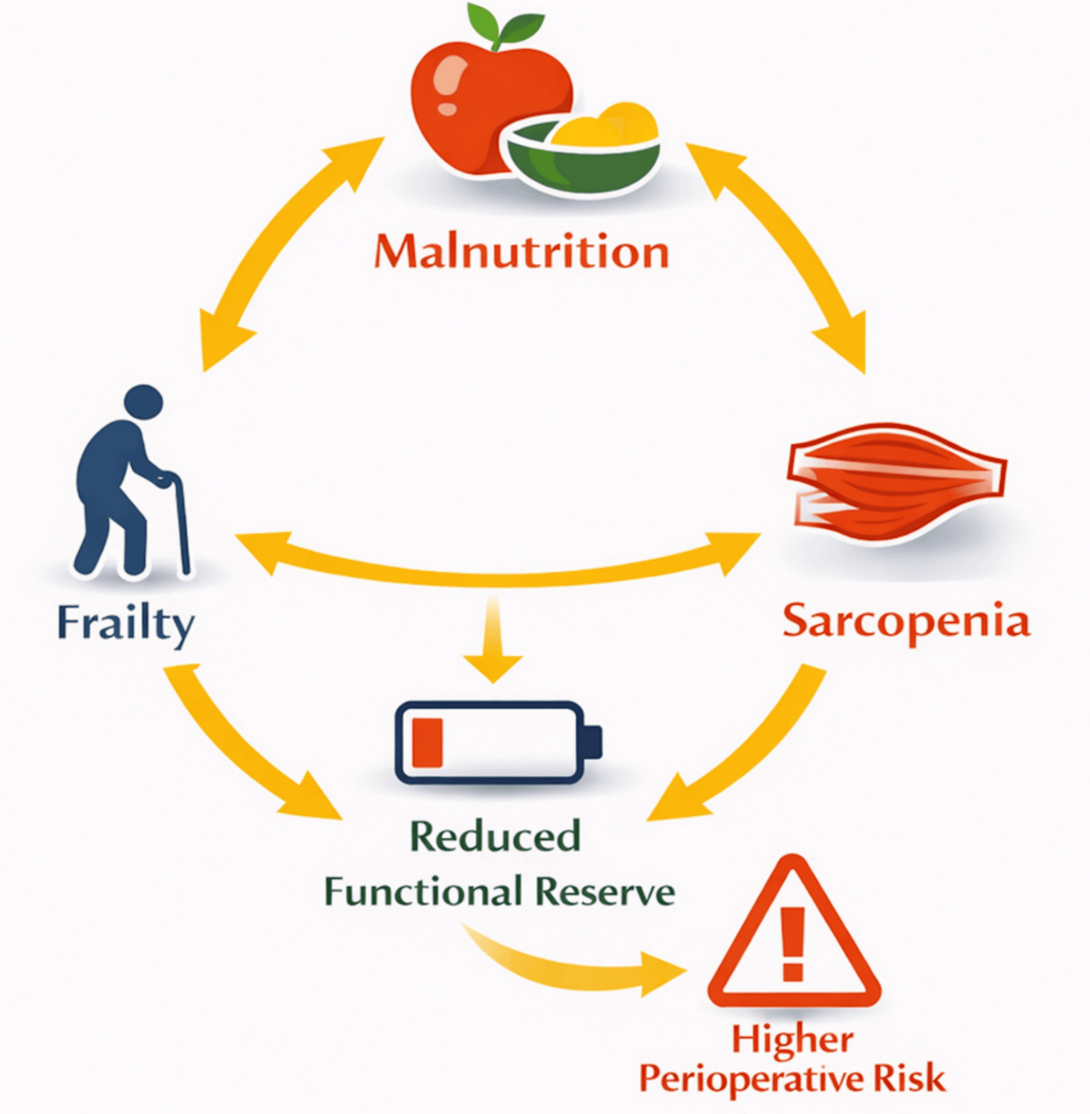
Circular vulnerability model in cardiovascular surgery This diagram demonstrates the interconnected relationship between frailty, malnutrition, and sarcopenia, which form a reinforcing triad commonly seen in high-risk cardiovascular surgery candidates. This figure was drawn with the help of BioRender.

Figure 2 illustrates the interdependent cycle linking frailty, malnutrition, and sarcopenia, and how this triad contributes to reduced functional reserve and higher perioperative risk in cardiovascular surgery patients.

Exercise training: Exercise training is the core component of most cardiac prehabilitation programs and aims to increase physiological reserve before surgery through targeted improvements in aerobic capacity, muscle strength, and respiratory function [7,12,34]. Contemporary reviews and meta-analyses indicate that exercise-based prehabilitation is generally safe, improves preoperative functional capacity, and may shorten postoperative length of stay and reduce complications in selected cardiovascular surgery populations [7,12,13].

Aerobic training: Aerobic training protocols in cardiac prehabilitation are usually modeled on phase II cardiac rehabilitation but compressed into the preoperative waiting period. Typical prescriptions include supervised or home-based walking, cycle ergometry, or treadmill training at moderate intensity (40-70% of heart rate reserve or Borg 11-14), delivered two to five times per week [7,12]. Some programs individualize intensity using CPET or six-minute walk performance, with progression toward interval or higher intensity bouts in fitter patients [7,13].

Randomized trials in patients awaiting CABG have shown that a two-week ambulatory prehabilitation program with walking and cycling improves preoperative six-minute walk distance and quality of life without increasing adverse events [35]. Emerging trials in frail cardiovascular surgery candidates suggest that twice-weekly supervised exercise can be delivered safely and leads to better early quality of recovery scores, particularly in those with higher baseline frailty [13,36]. Protocols under evaluation in ongoing studies commonly combine center-based supervised sessions with home exercise, reflecting the need to balance intensity with feasibility and travel burden [13,35-37].

Resistance and IMT: Resistance training is increasingly incorporated to address sarcopenia, low grip strength, and impaired functional mobility that are highly prevalent in older cardiovascular surgery patients [7,12]. Exercises usually target major lower-limb and upper-limb muscle groups using body weight, elastic bands, or light free weights, performed two or three times per week at 8-15 repetitions per set [7,12,35]. Evidence from coronary artery disease cardiac rehabilitation shows that adding resistance training to aerobic exercise yields small but significant gains in peak oxygen uptake and larger improvements in muscle strength and mobility, particularly in older adults [35]. These data support the inclusion of resistance work in prehabilitation, even though direct preoperative randomized data remain limited.

IMT is the best studied “resistance” modality in the preoperative setting. Multiple randomized trials and meta-analyses demonstrate that preoperative IMT, delivered with threshold loading devices at 30-60% of maximal inspiratory pressure, reduces postoperative pulmonary complications and shortens hospital stay after cardiovascular surgery [11,38-40]. Protocols vary in intensity and duration but often involve twice-daily sessions of 15-30 minutes over one to two weeks or short intensive regimens of three to five days when the waiting time is limited [11,38,39]. A recent systematic review focused on IMT in cardiovascular surgery confirms consistent improvements in inspiratory muscle strength, lung function, and reductions in pulmonary complication rates, with very low reported rates of training-related adverse events [40]. A contemporary randomized trial in elective valve surgery showed that even a three-day IMT program improved preoperative lung function and reduced postoperative pulmonary complications compared with sham or usual care [39].

Evidence for VO_2_ improvement: Although most cardiac prehabilitation trials have prioritized functional outcomes such as six-minute walk distance, emerging data suggest that structured exercise can also improve maximal oxygen uptake (VO_2_max) before surgery. In a recent systematic review and meta-analysis of exercise-based prehabilitation before cardiovascular surgery, a subset of trials that measured VO_2_max demonstrated small to moderate increases in aerobic capacity compared with usual care, alongside larger gains in walking distance and inspiratory muscle strength [13]. These findings align with broader cardiac rehabilitation literature, where postoperative supervised aerobic and combined aerobic plus resistance training in patients with coronary artery disease increases peak VO_2_ by approximately 0.7-1.0 mL·kg⁻¹·min⁻¹ and improves muscle strength and mobility [37,41]. A large meta-analysis of randomized trials across cardiovascular and metabolic conditions also confirms that structured exercise consistently raises cardiorespiratory fitness, with greater effects at higher weekly training volumes and intensities [42].

Importantly, even modest improvements in peak VO_2_ are prognostically meaningful. In patients with chronic heart failure, an increase in VO_2_peak over three months of training has been associated with better clinical outcomes, supporting the concept that small preoperative gains in aerobic capacity could translate into improved resilience to surgical stress [43]. From a mechanistic standpoint, these changes reflect peripheral adaptations in skeletal muscle oxidative capacity and endothelial function as well as central cardiovascular adaptations, all of which are relevant to the hemodynamic demands of cardiopulmonary bypass and early mobilization.

Duration of training required to modify physiology: The optimal duration of prehabilitation remains uncertain and is constrained by surgical waiting times. Most cardiac prehabilitation studies have implemented programs lasting two to eight weeks, with two to five supervised sessions per week plus home exercise [12,13,35]. A randomized trial of two-week ambulatory prehabilitation before coronary artery bypass surgery showed clinically important improvements in functional capacity and quality of life, demonstrating that meaningful adaptation can occur within a relatively short preoperative window [35]. Meta-analytic data from broader exercise training literature indicate that programs of 4-12 weeks are usually required to achieve maximal gains in VO_2_max and cardiometabolic biomarkers, although early adaptations can be observed within the first month [41,42].

Respiratory muscle training appears to require even shorter exposure. Meta-analyses and randomized trials suggest that IMT protocols as brief as three to seven days can significantly increase inspiratory muscle strength and reduce postoperative pulmonary complications [11,38,39]. This makes IMT particularly attractive for patients with limited lead time to surgery or those waiting in the hospital. However, for global improvements in aerobic capacity, strength, and frailty, longer multimodal programs are likely necessary, and current narrative and systematic reviews emphasize designing flexible pathways that can be scaled according to the time available before surgery [7,12,13].

Nutrition Optimization

Nutritional optimization is a central pillar of prehabilitation in cardiovascular surgery because malnutrition, sarcopenia, and low protein reserves are highly prevalent and strongly associated with postoperative complications and mortality [32,44,45]. Preoperative strategies focus on achieving adequate protein intake, meeting energy needs through caloric supplementation, and targeting patients who are already malnourished or at nutritional risk.

Protein targets: International perioperative nutrition guidelines recommend higher protein intakes for surgical patients than for healthy adults. The European Society for Clinical Nutrition and Metabolism (ESPEN) practical guideline on clinical nutrition in surgery advises a daily protein intake of approximately 1.2-2.0 g/kg body weight in patients at nutritional risk, with the upper end of the range used in severe catabolic states or in those with significant weight loss [44]. The ESPEN guideline on hospital nutrition similarly states that standard hospital diets should provide at least 1.2 g/kg/day of protein, with further increases and oral nutritional supplements for patients who are malnourished or unable to meet requirements by normal food intake [45].

In the context of cardiovascular surgery, these higher protein targets are particularly relevant because many patients are older, frail, and sarcopenic. Observational studies show that low preoperative protein and albumin or prealbumin levels correlate with increased infection rates, prolonged mechanical ventilation, and longer length of stay after cardiac procedures [32,46]. Achieving at least 1.2 g/kg/day of protein, often through a combination of diet and high protein oral supplements, is therefore recommended as a core objective of prehabilitation for nutritionally at-risk cardiac surgical patients [32,44-46].

Caloric supplementation: Energy provision in prehabilitation aims to prevent further weight loss and support protein anabolism. ESPEN surgical guidance suggests a total energy intake of roughly 25-30 kcal/kg/day in stable surgical patients, with 30-35 kcal/kg/day used in undernourished or highly stressed individuals when tolerated [44,47]. For many cardiovascular surgery candidates, habitual intake falls below these targets because of anorexia, breathlessness, early satiety, or hospital-related diet restrictions [32,48].

Caloric supplementation typically uses oral nutritional supplements (ONS) that provide both energy and protein. These are prescribed one to three times per day in addition to regular meals, with composition tailored toward high-protein, high-energy formulations when weight loss or low body mass index is present [44,45]. A systematic review of oral and enteral nutritional support in adult cardiovascular surgery found that preoperative nutrition interventions, particularly carbohydrate loading drinks and energy-dense supplements, improved perioperative metabolic responses and occasionally reduced length of stay, although effects on mortality and intensive care stay were inconsistent across heterogeneous trials [48].

Even when definitive outcome benefits are modest, perioperative nutrition experts highlight that supplemental calories are necessary to achieve recommended protein targets without provoking further catabolism, especially when patients participate in exercise-based prehabilitation programs that increase energy expenditure [44,47,48].

Outcomes in malnourished cardiac patients: A substantial body of observational evidence links poor preoperative nutritional status with adverse outcomes after cardiovascular surgery. In a large prospective cohort of patients undergoing cardiopulmonary bypass, Lomivorotov et al. showed that malnutrition identified by several screening tools was independently associated with higher rates of complications, prolonged intensive care stay, and increased hospital mortality [32].

Biochemical markers of protein-energy malnutrition also carry prognostic value. A study of more than 600 adult cardiovascular surgery patients reported that a preoperative prealbumin level of 20 mg/dL or less was associated with significantly higher risk of postoperative infection and longer duration of mechanical ventilation [46]. More recent work in older adults undergoing valve surgery demonstrated that objective nutritional scores such as the Geriatric Nutritional Risk Index and Controlling Nutritional Status score independently predicted one-year mortality and major complications, even after adjustment for traditional surgical risk scores [48].

Similar findings have been reported in broader adult cardiovascular surgery cohorts, where malnutrition assessed by various anthropometric and biochemical measures was associated with longer hospital stays, higher rates of acute kidney injury, and increased in-hospital mortality [47,49]. A contemporary prospective study found that nearly half of preoperative hospitalized cardiovascular surgery patients met criteria for malnutrition and that poorer nutritional status correlated with worse postoperative outcomes and reduced quality of life [49].

These data indicate that malnutrition is common among cardiovascular surgical candidates and is a modifiable risk factor that should be systematically addressed within prehabilitation. Identifying patients at nutritional risk through screening tools and laboratory markers, then delivering targeted protein-rich and energy adequate nutritional support, has the potential to reduce complications and support functional recovery in this high-risk population [32,44-49].

Smoking and Alcohol Cessation

Tobacco and hazardous alcohol use are among the most important modifiable risk factors addressed in prehabilitation for cardiovascular surgery. Both are strongly associated with higher rates of pulmonary, cardiovascular, and wound complications, as well as longer hospital stay and mortality after major surgery [50-56]. While most evidence comes from mixed surgical cohorts, current cardiac prehabilitation reviews endorse applying these principles to patients awaiting coronary, valve, and aortic procedures [7].

Smoking cessation: Smokers have substantially higher risks of postoperative pulmonary complications, wound infection, and cardiovascular events compared with non-smokers [51-53]. A landmark randomized trial by Møller et al. showed that an intensive smoking cessation program starting six to eight weeks before elective hip and knee arthroplasty halved the overall postoperative complication rate compared with usual care [51]. Subsequent Cochrane and narrative reviews confirm that preoperative smoking interventions combining counseling with nicotine replacement therapy increase quit rates at the time of surgery and at 12 months and probably reduce postoperative morbidity [52].

With regard to the minimum abstinence period, a large systematic review and meta-analysis by Mills et al. found that preoperative smoking cessation reduced overall postoperative complications, with longer periods of abstinence associated with larger benefits. Trials with at least four weeks of cessation had significantly greater risk reduction than shorter programs, and each additional week of abstinence before surgery improved outcomes by roughly 19% [50]. Wong et al. reported that quitting smoking more than four weeks before surgery reduced respiratory complications and that abstinence of at least three to four weeks reduced wound healing complications, whereas cessation within the preceding four weeks did not increase respiratory risk compared with continued smoking [57].

The 2020 WHO Tobacco Knowledge Summary concludes that quitting smoking approximately four weeks before surgery lowers the risk of complications and that further gains occur with longer abstinence, while also emphasizing that even short-term cessation is preferable to continued smoking [1]. Contemporary perioperative guidance therefore recommends advising all cardiovascular surgery candidates who smoke to stop as early as possible, ideally eight or more weeks preoperatively, with a minimum target of at least four weeks of abstinence before elective procedures [7,50,52,53,57]. Importantly, there is no high-quality evidence that quitting shortly before surgery increases perioperative risk, so patients should still be encouraged to stop even when surgery is imminent [53,57].

Alcohol cessation: High-risk alcohol consumption is independently associated with increased postoperative infections, cardiopulmonary complications, bleeding, and prolonged hospital stay [54-56]. In a randomized controlled trial of alcohol misusers undergoing various elective operations, one month of complete preoperative abstinence achieved through a structured intervention significantly reduced postoperative morbidity compared with continued drinking [54].

The Cochrane review by Oppedal et al., subsequently updated by Egholm et al., identified three randomized trials of intensive perioperative alcohol cessation interventions in hazardous drinkers [55,56]. Programs typically combined pharmacologic management of withdrawal, counseling, and relapse prevention and aimed for complete abstinence for four to eight weeks before surgery. Pooled data suggested that such intensive interventions probably reduce postoperative complications, although sample sizes were small and all trials were conducted in Scandinavian populations [56].

Based on these data, international perioperative guidance generally recommends a minimum of four weeks of complete abstinence for patients with risky alcohol use, with six to eight weeks considered optimal when feasible [54-56]. For cardiovascular surgery, where cardiopulmonary reserve is already limited, screening for hazardous drinking and offering structured cessation support during the prehabilitation period are particularly important. Even when the full recommended abstinence window cannot be achieved, any reduction in alcohol intake before surgery is likely to confer some risk reduction and should be encouraged [53-56].

Psychological Preparation

Psychological preparation is a core component of prehabilitation for cardiovascular surgery, since anxiety and mood symptoms are highly prevalent and directly influence perioperative physiology, pain, and engagement with recovery behaviors [58]. Preoperative anxiety has been associated with higher postoperative pain scores, greater opioid requirements, delayed mobilization, and longer hospital stay, including in cardiovascular surgery cohorts [59,60]. At the same time, depression is common in patients with cardiovascular disease and is linked to poorer self-management and higher mortality [61,62]. A structured approach that combines screening, targeted psychological interventions, and high-quality patient education is therefore central to contemporary prehabilitation models.

Anxiety reduction: Preoperative anxiety affects a large proportion of surgical patients, with estimates in reviews ranging from 40% to 80% depending on population, procedure, and measurement tools [59,63]. In a cohort of patients undergoing cardiovascular surgery, moderate to severe preoperative anxiety was associated with significantly higher postoperative pain scores and increased intraoperative and postoperative morphine use compared with patients with only mild anxiety [60]. A systematic review and meta-analysis by Shebl et al. found that preoperative anxiety was correlated with higher anesthetic and analgesic requirements, prolonged time to extubation, and increased risk of postoperative delirium in adults, underscoring its physiologic impact [64].

Psychological preparation aims to attenuate this anxiety through information provision, coping skills training, and relaxation techniques. The classic Cochrane review of psychological preparation for adults undergoing surgery and invasive procedures showed that such interventions reduce negative affect and may improve pain, behavioral recovery, and length of stay across a range of operations [58]. More recent nursing-led interventions in cardiac units have applied these principles through structured preoperative sessions that combine education with breathing exercises, cognitive reframing, and reassurance. In a randomized controlled trial of mechanically ventilated cardiac patients, preoperative education and coaching reduced anxiety scores, improved hemodynamic stability, and enhanced patient comfort compared with standard care [65]. Similarly, a quasi-experimental study in patients undergoing CABG reported that a short preoperative training program delivered by nurses significantly improved postoperative comfort scores [61]. Collectively, these data support routine screening for anxiety and the incorporation of brief, scalable psychological interventions within prehabilitation pathways.

Depression risk and impact on adherence: Depressive symptoms are present in up to one-third of patients with established cardiovascular disease and are consistently associated with adverse outcomes, including rehospitalization and mortality [62,66]. A comprehensive review by Goldstein et al. highlighted that depression is tightly linked to medication nonadherence and poorer engagement with recommended health behaviors such as smoking cessation, physical activity, and participation in cardiac rehabilitation [62]. Importantly for prehabilitation, depression appears to impair the very behaviors that underlie successful conditioning before surgery, including regular exercise, nutritional optimization, and attendance at education sessions.

Prospective data suggest that improving depressive symptoms can enhance adherence. In a randomized trial of collaborative care for depressed cardiac inpatients, Bauer et al. found that reductions in depression scores over six months were independently associated with better self-reported adherence to medications and secondary prevention behaviors [67]. These findings indicate that psychological preparation for cardiovascular surgery should not be limited to anxiety management but should include systematic screening for depression, brief evidence-based interventions (such as problem-solving therapy or cognitive behavioral strategies), and close coordination with mental health services when more intensive treatment is required. Addressing depression early may improve engagement with prehabilitation tasks and, downstream, affect postoperative recovery.

Patient education: Patient education is the most visible component of psychological preparation and is embedded in many cardiac prehabilitation programs. Education can normalize fears, correct misconceptions about surgery and anesthesia, and enhance patients’ sense of control, which in turn reduces anxiety and improves satisfaction [58,63]. In a systematic review focused on cardiovascular surgery, Guo identified six randomized trials of preoperative education and reported mixed but generally favorable effects on psychosocial and physical recovery; several trials showed reductions in anxiety and improvements in mood and functional outcomes, whereas others found neutral effects [68].

More recent work has refined both content and delivery. Pazar and Iyigun conducted a randomized controlled trial in cardiac patients and showed that individualized preoperative education that covered the ICU environment, mechanical ventilation, expected symptoms, and coping strategies significantly reduced anxiety and improved comfort and patient-ventilator synchrony compared with usual care [65]. Şahin and Dığın reported that a structured training session before CABG improved postoperative comfort scores on the General Comfort Questionnaire, supporting the value of relatively brief, nurse-delivered interventions [61]. Beyond traditional verbal and written formats, immersive technologies are being tested. In the Virtual Reality (VR) Patient Journey Trial, El Mathari et al. randomized cardiovascular surgery patients to virtual-reality-based education or standard information and found that although VR did not reduce preoperative anxiety scores, it significantly increased patient satisfaction with the information received and perceived preparedness for surgery [69].

Narrative and systematic reviews of preoperative patient education across surgical specialties concur that education usually lowers anxiety, though effects vary by content, timing, and patient characteristics [63,70]. Samadi’s recent literature review emphasized that most studies show a meaningful reduction in preoperative anxiety with education, but some report neutral or even increased anxiety when information is poorly tailored or overly technical [59]. These observations are highly relevant for cardiovascular prehabilitation: educational materials must be culturally and linguistically adapted, sensitive to health literacy, and focused on practical expectations and coping strategies rather than graphic procedural detail. Multimodal programs that integrate face-to-face discussion, written materials, videos, and possibly VR, delivered by an interdisciplinary team, appear most promising for optimizing psychological readiness before major cardiovascular surgery.

Optimization of Comorbidities

Optimizing medical comorbidities before cardiovascular surgery is a core aim of prehabilitation because baseline illness burden strongly predicts perioperative risk. Diabetes, anemia, and chronic pulmonary disease remain among the most influential modifiable factors in this population. Effective preoperative medication management also plays a central role in reducing surgical complications and improving physiological readiness.

Diabetes management: Diabetes affects up to 40% of cardiovascular surgery patients and is independently associated with infection, renal dysfunction, and mortality [71]. Elevated preoperative HbA1c correlates with prolonged intensive care stay and increased postoperative mortality after major surgery [72]. Current diabetes optimization strategies include early detection of poor glycemic control, medication review, and targeted perioperative glucose management [73]. Professional guidance recommends continuing basal insulin, avoiding extreme hyperglycemia or hypoglycemia, and coordinating endocrinology input for patients with poorly controlled disease prior to elective surgery [73].

Iron-deficiency anemia treatment: Preoperative anemia is common in candidates for valve or coronary procedures and substantially increases postoperative risk. A 2022 review noted that anemia in cardiac surgical patients increases transfusion rates, acute kidney injury, and length of stay [74]. Optimization begins with early hemoglobin screening, iron studies, and treatment of iron deficiency using oral or intravenous supplementation, depending on urgency [74]. Meta-analyses in mixed surgical cohorts show that correcting iron deficiency improves hemoglobin levels and may reduce transfusion exposure and complications [75]. Treating anemia during prehabilitation complements exercise and nutritional interventions by supporting muscle oxygen delivery and recovery capacity.

Pulmonary disease optimization: Chronic obstructive pulmonary disease (COPD) and other chronic lung conditions strongly influence postoperative respiratory complications in cardiovascular surgery [76]. COPD severity correlates with increased mortality, prolonged ventilation, and pneumonia risk following coronary artery bypass grafting [76]. Pulmonary optimization includes inhaled bronchodilator adjustment, smoking cessation, treatment of infection or exacerbation, respiratory physiotherapy, and IMT where feasible [77]. These interventions aim to reduce postoperative pulmonary morbidity and improve functional performance before surgery.

Medication tuning prior to surgery: Medication review is another critical component of prehabilitation. Continuation of beta-blockers in patients with established indications reduces arrhythmic and ischemic events around cardiovascular surgery [71]. Statin therapy is generally continued because it lowers cardiovascular events and may improve graft patency [71]. When patients take antiplatelet or anticoagulant drugs, particularly those with prior stents or atrial fibrillation, therapy must be carefully timed and adjusted around surgery to reduce bleeding risk without compromising cardiac protection [73,78].

Table 2 provides an overview of the core components of multimodal prehabilitation in cardiovascular surgery, summarizing the key interventions, the relative strength of evidence, and the primary outcomes associated with each domain.

**Table 2:** Prehabilitation components and their evidence base in cardiovascular surgery Evidence strength ratings are based on a qualitative author assessment of the available literature, considering the volume, consistency, and methodological quality of evidence, including the presence of randomized controlled trials, systematic reviews, and meta-analyses where available. VO_2_ peak: Peak oxygen consumption; PPCs: Postoperative pulmonary complications; ICU LOS: Intensive care unit length of stay; 1RM: One-repetition maximum; MIP: Maximal inspiratory pressure; CFS: Clinical frailty scale; NRT: Nicotine replacement therapy; AUDIT: Alcohol use disorders identification test; COPD: Chronic obstructive pulmonary disease; HF: Heart failure.

Representative Studies	Component	Key Interventions	Evidence Strength	Main Outcomes
Arthur et al., 2000 [79]	Aerobic exercise training	Walking, cycling, treadmill training; interval or steady-state sessions	Moderate–high	↑ VO_2_ peak, ↑ functional reserve, ↓ postoperative complications, ↓ length of stay
Tew et al., 2022 [80]	Resistance training	Strengthening of major muscle groups 2–3×/week; 60%–80% 1RM; 8–12 repetitions	Moderate	↑ muscle strength, ↑ mobility, ↓ sarcopenia progression
Neto et al., 2017 [81]	Inspiratory muscle training (IMT)	Threshold loading 30%–60% MIP; daily sessions for 1–4 weeks	High	↓ PPCs, ↓ pneumonia, ↓ ventilator time, ↓ ICU LOS
Weimann et al., 2021 [44]	Nutritional optimization	Protein optimization (1.2–2.0 g/kg/day), dietitian counseling, caloric optimization	Moderate	↑ nutritional status, ↓ infections, ↓ complications
Afilalo et al., 2017 [82]; McIsaac et al., 2020 [83]	Frailty assessment and optimization	CFS, Fried phenotype, gait speed, grip strength; combined nutrition + exercise	Moderate	Improved risk prediction, ↓ postoperative complications
Tully et al., 2008 [84]; Powell et al., 2016 [58]	Psychological preparation	Anxiety/depression screening, coping skills, relaxation, pre-op education, VR	Low–moderate	↓ anxiety, ↑ patient engagement, ↑ surgical readiness
Mills et al., 2011 [50]; Wong et al., 2012 [57]	Smoking cessation	Counseling, NRT, behavioral therapy; ≥4–8 week abstinence	High	↓ pulmonary and wound complications, ↓ LOS
Oppedal et al., 2012 [55]	Alcohol reduction	6–8 week abstinence, behavioral support, AUDIT screening	Moderate	↓ infections, ↓ bleeding, ↓ LOS
De Hert et al., 2018 [85]	Comorbidity optimization	Glycemic control, anemia management, COPD optimization, HF management	Moderate–high	↓ complications, ↑ physiological reserve

Physical functional outcomes

VO_2_ Max Improvement

VO_2_ max is an established marker of aerobic capacity and surgical resilience. Although cardiac-specific prehabilitation trials remain limited, recent systematic reviews have shown that structured preoperative aerobic and multimodal training can improve peak oxygen uptake before major surgery, including cardiac operations [13]. In a comprehensive systematic review and meta-analysis of exercise-based prehabilitation in adult cardiovascular surgery patients, Hurtado-Borrego et al. identified measurable gains in VO_2_ max across the subset of randomized trials that included aerobic capacity as an outcome, indicating meaningful improvement in cardiorespiratory fitness prior to surgery [13]. Similar patterns have been documented in outpatient cardiac rehabilitation, where combined aerobic and resistance training produced significant increases in VO_2_ peak in patients with coronary artery disease [37]. These data suggest that appropriately dosed exercise training during prehabilitation can produce short-term physiological adaptations relevant to surgical stress tolerance.

Six-minute walk distance (6MWD): The six-minute walk distance is the most commonly reported functional metric in cardiac prehabilitation studies. A randomized controlled trial in coronary artery bypass graft candidates showed that a two-week ambulatory prehabilitation program significantly increased preoperative 6MWD and improved quality of life compared with usual care [35]. A 2023 meta-analysis of cardiac prehabilitation trials demonstrated a consistent increase in 6MWD among intervention groups, supporting its role as a sensitive outcome measure for aerobic and musculoskeletal improvement before surgery [13]. Additional evidence from frailty-focused cardiac trials indicates that structured exercise programs improve functional mobility and early postoperative recovery scores [36].

Hand-grip strength: Hand-grip strength reflects global muscle function and correlates with frailty and postoperative outcomes in cardiovascular surgery. Observational evidence shows that low preoperative grip strength is associated with longer intensive care stay and higher postoperative complication rates [86]. While fewer trials evaluate grip strength change directly, multimodal prehabilitation programs incorporating resistance exercise have demonstrated significant improvements in upper-limb muscle force in coronary artery disease populations undergoing structured training [37]. These findings suggest that targeted muscle conditioning during prehabilitation may enhance perioperative strength and support faster postoperative mobilization.

Clinical Outcomes

Evidence for the impact of prehabilitation on clinical outcomes in cardiovascular surgery is growing but remains heterogeneous. Most studies are small single-center trials or observational cohorts, and many are underpowered for hard endpoints such as mortality, yet consistent patterns are emerging for length of stay, postoperative complications, and some process measures.

Length of stay and ICU time: Several cardiac-focused trials and reviews suggest that prehabilitation can reduce postoperative hospital stay and, in some cases, ICU time. In a systematic review of preparative rehabilitation for adult cardiovascular surgery, Yau et al. reported that most included trials showed shorter total length of stay in intervention groups, although the magnitude of reduction varied and confidence intervals often crossed unity because of small sample sizes [9]. A meta-analysis of preoperative IMT in cardiovascular surgery found a significant reduction in postoperative hospital stay of approximately one to two days, driven largely by fewer pulmonary complications and earlier readiness for discharge [11].

Randomized controlled trials support these findings. Chen et al. demonstrated that five days of intensive preoperative IMT before elective cardiovascular surgery reduced postoperative pulmonary complications and shortened both ICU and overall hospital stay compared with usual care [10]. Similarly, a two-week multimodal prehabilitation program for patients awaiting CABG improved functional capacity and showed a trend toward reduced length of stay, although the study was not powered for this endpoint [35].

Readmission and complication rates: Readmission has been less frequently studied. In the cardiac prehabilitation review by Yau et al., only a minority of trials reported readmission data, and most did not find statistically significant differences, reflecting limited power rather than a clear absence of effect [9]. In contrast, perioperative risk factor modification studies, particularly smoking and alcohol cessation trials that are commonly considered part of multimodal prehabilitation, provide stronger evidence for complication reduction.

A systematic review and meta-analysis of preoperative smoking cessation found that patients who quit before surgery had significantly fewer total postoperative complications, including wound and pulmonary events, compared with those who continued to smoke [50]. In a landmark randomized trial, an intensive preoperative smoking intervention starting six to eight weeks before elective orthopedic surgery more than halved overall complication rates and reduced wound problems [51]. Likewise, a randomized trial of patients with hazardous alcohol use showed that one month of complete preoperative abstinence substantially reduced postoperative morbidity across several organ systems [54]. Although these studies were not restricted to cardiovascular surgery, the mechanisms they address, such as improved immune function and respiratory status, are directly relevant to cardiovascular operations.

In the cardiac-specific IMT meta-analysis, prehabilitation significantly reduced postoperative pulmonary complications, including pneumonia, atelectasis, and respiratory failure, which are major drivers of ICU utilization and length of stay in this population [11]. Chen et al. reported fewer pulmonary complications and earlier extubation in the IMT group, further supporting a protective effect against respiratory morbidity [10].

Postoperative delirium: Postoperative delirium is a common and serious complication in older cardiovascular surgery patients, associated with longer hospital stay, institutionalization, and mortality. Direct evidence that prehabilitation reduces delirium is limited, but several components of prehabilitation address established risk factors such as frailty, poor functional reserve, and baseline cognitive vulnerability. In older surgical patients, low functional capacity and preoperative cognitive impairment have been linked to a higher risk of delirium and worse long-term outcomes [87]. By improving aerobic capacity, muscle function, and psychological readiness, prehabilitation may indirectly reduce delirium risk, a hypothesis that warrants targeted evaluation in future cardiac-specific trials.

Mortality: Mortality has rarely been a primary endpoint in prehabilitation research. In the cardiovascular surgery systematic review by Yau et al., most included studies reported no significant difference in short-term mortality between prehabilitation and usual care, but they were underpowered for this outcome [9]. The IMT meta-analysis similarly found no clear effect on mortality, despite improvements in pulmonary complications and length of stay [11]. Large multicenter trials with longer follow-up will be needed to determine whether the cumulative reduction in morbidity and improved functional reserve achieved through multimodal prehabilitation can translate into survival benefits in high-risk cardiovascular surgical populations.

Patient-Reported Outcomes

Psychological and health-related quality of life (HRQoL) outcomes are central to evaluating prehabilitation effectiveness because functional gains alone do not capture the lived experience of cardiovascular surgery patients. Prehabilitation programs commonly include education, psychological support, and structured exercise, all of which target anxiety, confidence, symptom burden, and patient-perceived recovery.

Anxiety: Prehabilitation consistently demonstrates reductions in preoperative anxiety. In a randomized controlled trial of patients awaiting cardiovascular surgery, Pazar and Iyigun found that structured preoperative education significantly reduced anxiety scores and improved comfort compared with usual care [65]. A systematic review evaluating nursing-led anxiety interventions before surgery similarly showed significant reductions in anxiety and greater emotional preparedness among intervention cohorts [63]. These findings support the concept that early engagement, information delivery, and coping strategies help reduce affective distress before cardiovascular surgery.

Quality of life: Several trials have shown that prehabilitation improves HRQoL before and after cardiovascular surgery. In patients awaiting CABG, a randomized controlled trial demonstrated that two weeks of multimodal prehabilitation enhanced both physical and mental quality-of-life scores measured by validated instruments [35]. Comparable improvements were seen in a randomized cardiac prehabilitation study assessing postoperative recovery, where patients reported better health status and symptom control following structured exercise and support programs [35]. These effects likely reflect increased functional reserve, improved mood, and greater treatment confidence.

Return to baseline function: Patient-reported functional recovery, including return to baseline activities, is becoming an important metric in perioperative research. A review of preparatory rehabilitation in cardiovascular surgery noted that participants receiving prehabilitation reported quicker perceived return to normal activity and daily function, aligning with objective improvements in exercise capacity and postoperative mobility [9]. Although long-term follow-up data remain limited, the available evidence suggests that multimodal programs accelerate subjective recovery trajectories and may reduce disability time.

Special Populations

Certain patient groups undergoing cardiovascular surgery may derive particular benefit from prehabilitation because they carry disproportionate perioperative risk. Emerging literature suggests potential advantages for elderly, frail, female, obese, and transplant-listed individuals, although the strength of evidence varies across these domains.

Elderly patients: Older adults represent the majority of cardiac surgical candidates and show higher rates of functional decline and postoperative complications. Prehabilitation is especially relevant because age-related reductions in aerobic capacity and muscle mass limit reserve to withstand surgical stress. A systematic review demonstrated that exercise-based cardiac prehabilitation improved physical capacity and early postoperative recovery outcomes in older patients, supporting its suitability for geriatric cardiac cohorts [13]. Preoperative assessment tools in elderly cardiovascular surgery patients, including gait speed, grip strength, and comprehensive geriatric evaluation, help identify those who may benefit most from targeted conditioning [19].

Frail patients: Frailty strongly predicts mortality, prolonged hospitalization, and institutionalization after cardiovascular surgery. A study of advanced heart failure patients referred for transplant evaluation showed that frailty was independently associated with worse survival outcomes and poorer functional status [19]. Because frailty reflects multisystem vulnerability, multimodal prehabilitation targeting physical reconditioning, nutrition, and psychological resilience may mitigate perioperative deterioration. Preliminary cardiac studies demonstrate that preoperative exercise interventions improve functional metrics in frail individuals, although large controlled trials remain limited [13].

Women: Women undergoing cardiovascular procedures may present with greater symptom burden, smaller body size, and later disease stage than men. Postoperative recovery trajectories are also slower in women due to differences in muscle mass and inflammatory response. A meta-analysis of preoperative IMT in cardiovascular surgery included sex-balanced populations and demonstrated significant reductions in pulmonary complications and length of stay, suggesting that women may experience meaningful physiologic benefit from prehabilitation [11]. Further research is needed to explore sex-specific training adaptations and psychosocial barriers to program access.

Obese patients: Obesity is associated with increased risk of wound infection, arrhythmia, and respiratory complications following cardiac operations. Evidence from coronary artery disease rehabilitation cohorts indicates that structured resistance and aerobic training can improve body composition and exercise tolerance in obese cardiac patients [37]. Prehabilitation may enhance postoperative mobility, respiratory mechanics, and glycemic control in this group. Nutrition counseling combined with medical weight management may further improve surgical readiness, although cardiac-specific trials remain scarce.

Transplant candidates: Patients awaiting heart transplantation represent a highly vulnerable subgroup with extreme deconditioning and prolonged hospitalization risk. A study examining frailty among heart-transplant candidates showed a strong association between frailty and increased mortality and morbidity while on transplant waitlists [19]. Prehabilitation strategies, including strength training, inspiratory muscle work, and nutritional optimization, are increasingly recognized as necessary to preserve muscle mass and cardiorespiratory reserve while awaiting surgery. Early pilot data suggest these programs improve exercise tolerance and may reduce postoperative disability, but randomized studies are still needed.

It should be noted that cardiac prehabilitation trials have rarely been specifically designed to enroll or stratify patients according to subgroups such as sex, obesity, or transplant status, and subgroup-specific evidence therefore remains limited. Table 3 summarizes the reported effects of prehabilitation on key physiological, functional, and clinical outcomes in patients undergoing cardiovascular surgery, highlighting the direction and consistency of observed benefits across studies.

**Table 3:** Summary of prehabilitation outcomes in cardiovascular surgery * denotes a study of prehabilitation in abdominal surgery (not cardiovascular surgery). VO_2_ peak: Peak oxygen consumption; 6MWD: 6-minute walk distance; ICU: Intensive care unit; QoL: Quality of life.

Key Supporting Evidence	Outcome Domain	Direction of Effect with Prehabilitation	Consistency of Evidence	Clinical Relevance
Arthur et al., 2000 [79]; Tew et al., 2022 [80]	VO_2_ peak	Modest improvement	Moderate	Improved aerobic capacity and physiological reserve
Arthur et al., 2000 [79]; Barberan-Garcia et al., 2018* [88]	6-minute walk distance (6MWD)	Improvement	Moderate–high	Marker of functional capacity and recovery potential
McIsaac et al., 2020 [83]; Afilalo et al., 2017 [82]	Preoperative functional reserve	Improvement	Moderate	Improved tolerance to surgical stress
Hulzebos et al., 2006 [89]; Neto et al., 2017 [81]	Pulmonary complications	Reduction	High (IMT strongest)	Reduced pneumonia and respiratory morbidity
Hulzebos et al., 2006 [89]; Barberan-Garcia et al., 2018 [88]	ICU and ward length of stay	Reduction	Moderate	Faster postoperative recovery
Arthur et al., 2000 [79]; McIsaac et al., 2020 [83]	Mortality	No consistent reduction	Low	Studies underpowered for mortality endpoints
Powell et al., 2016 [58]; Tully et al., 2008 [84]	Patient-reported outcomes (QoL, anxiety)	Improvement	Low–moderate	Improved perioperative experience and engagement

Safety, Feasibility, and Barriers to Adoption

Prehabilitation for cardiovascular surgery is generally well tolerated and safe, but its routine implementation faces practical, economic, and logistical challenges. Available trials report low adverse event rates and high protocol adherence, indicating that structured exercise, breathing training, and education can be delivered safely to high-risk cardiac populations [13]. However, health system constraints and patient-level barriers continue to limit widespread adoption.

Time limitations before elective surgery: A major feasibility limitation is the short interval between the surgical decision and the operative date. Many cardiac patients undergo surgery within weeks of referral, restricting opportunities to achieve meaningful physiologic adaptation [35]. The elective waiting period varies widely between centers and healthcare systems, and patients with unstable angina or severe symptomatic valve disease often require expedited scheduling. These timelines limit the ability to deliver programs long enough to improve aerobic capacity and muscle mass, which typically require several weeks of stimulus [11].

Cost and resource limitations: Prehabilitation demands staffing, monitoring systems, exercise equipment, and institutional coordination. Hospitals may lack dedicated personnel or funding to deliver standardized programs, especially in regions without reimbursement structures [9]. A systematic review found that cardiac prehabilitation studies frequently cited financial and staffing limitations as obstacles to long-term implementation, regardless of program efficacy [13].

Patient motivation and adherence: Motivation is also a central challenge. Symptoms such as fatigue, dyspnea, and anxiety, which are common among cardiac patients, reduce exercise engagement [19]. Psychosocial stress, fear of surgery, and limited confidence in exercise safety can impede adherence. Studies emphasize that individualized education, psychological support, and early coaching improve uptake and completion rates [65].

Referral pathway problems: Referral processes remain inconsistent. Many cardiac patients are evaluated by multiple services, including cardiology, cardiovascular surgery, and primary care, without a unified referral workflow to prehabilitation services [9]. Lack of standardized guidelines results in variable program entry, reliance on clinician preference, and delayed initiation [87].

Cultural and socioeconomic barriers: Access inequities also limit participation. Lower socioeconomic status is associated with reduced involvement in rehabilitation and prehabilitation programs due to travel distance, transport cost, employment constraints, and limited health literacy [90]. Cultural perceptions of exercise and limited language-appropriate materials further hinder engagement. Addressing these barriers is critical for equitable program delivery.

Implementation Models

Hospital-based supervised programs: Traditional prehabilitation models involve center-based supervised training, allowing real-time monitoring and rapid progression. Trials using hospital-based exercise and IMT before cardiovascular surgery have demonstrated safety and feasibility with excellent retention rates [11]. Such programs are beneficial for frail or high-risk patients requiring close supervision, but scalability is limited by cost, geographic access, and staffing requirements [9].

Home-based tele-prehabilitation: Telemedicine-supported home-based prehabilitation models have emerged to address logistical and socioeconomic limitations. Reviews of home and telehealth cardiac rehabilitation demonstrate comparable improvements in functional outcomes and HRQoL to center-based care, with higher accessibility and lower resource burden [91]. These principles are now being applied to prehabilitation. Digital platforms, wearable monitors, and remote coaching allow individualized exercise progression and education without routine hospital attendance [91]. Early tele-prehabilitation pilots suggest good safety and adherence, offering a potential pathway to scale integration into routine cardiac care. Table 4 compares the principal models of prehabilitation delivery in cardiovascular surgery, highlighting their respective advantages, limitations, resource requirements, and supporting evidence.

**Table 4:** Models of prehabilitation delivery in cardiovascular surgery

Studies	Model of delivery	Advantages	Limitations	Resource needs
Arthur et al., 2000 [79]; Hulzebos et al., 2006 [89]	Center-based prehabilitation	Direct supervision, high safety, precise exercise prescription, and multidisciplinary input	Limited accessibility, travel burden, and higher costs	Dedicated facilities, trained staff, and monitoring equipment
Tew et al., 2022 [80]	Hybrid prehabilitation	Combines supervision with flexibility, improved adherence, and balanced resource use	Requires coordination and heterogeneous implementation	Initial center visits plus home training and tele-follow-up
McIsaac et al., 2020 [83]	Digital/tele-prehabilitation	Maximizes access, suitable for remote or frail patients, scalable, and supports monitoring	Digital literacy barriers, limited real-time supervision, and data integration issues	Wearables, mobile apps, telehealth platforms, and clinician oversight

Comparison with other surgical specialties

Although the evidence base for cardiovascular prehabilitation remains smaller compared to other surgical fields, outcomes reported in orthopedic, colorectal, and thoracic surgery reinforce the feasibility and expected benefit of structured preoperative conditioning. These specialties provide larger and more mature datasets, demonstrating that short-term, targeted interventions can meaningfully improve postoperative recovery, reduce complications, and enhance quality of life.

Orthopedic surgery: Prehabilitation is well established in joint replacement pathways. Randomized controlled trials in total knee arthroplasty have shown that structured preoperative exercise programs improve postoperative function and shorten hospital stay [92]. A systematic review of prehabilitation in hip and knee arthroplasty found significant improvements in quadriceps strength, mobility, and patient-reported outcomes, particularly when resistance training was incorporated [93]. These findings support the idea that meaningful physiological adaptation can occur within weeks and are directly relevant to cardiovascular surgery patients, who similarly experience frailty, sarcopenia, and exercise intolerance.

Colorectal surgery: Colorectal prehabilitation is among the best studied models and demonstrates clear physiologic and clinical benefits. A landmark randomized trial of multimodal prehabilitation in colorectal cancer surgery showed superior postoperative functional walking capacity compared with standard care, despite a shorter intervention period [94]. Multimodal colorectal protocols have also been associated with fewer complications and shorter length of stay [95]. Because colorectal patients share comparable age and comorbidity profiles with cardiac cohorts, these data reinforce the expectation that cardiovascular prehabilitation can produce similar improvements in exercise tolerance, complication rates, and postoperative mobilization.

Thoracic surgery: Thoracic surgery provides the closest analog for cardiac care because of shared cardiopulmonary physiology and pulmonary complication risk. Meta-analyses of IMT and exercise programs before lung resection have demonstrated reduced pulmonary complications, shorter hospital stays, and improved functional capacity [96]. Thoracic studies consistently highlight feasibility and safety, even in frail or older patients undergoing high-risk procedures [97]. These results parallel the emerging cardiac evidence base, particularly around IMT and aerobic conditioning, strengthening the anticipation of similar postoperative gains in cardiovascular surgery.

Evidence gaps

Although prehabilitation research in cardiovascular surgery has expanded, several important limitations persist. First, randomized controlled trials remain scarce. Most cardiac prehabilitation studies are single-center and underpowered, preventing clear conclusions regarding hard endpoints such as mortality, readmission, and long-term functional recovery [13]. Meta-analyses consistently report that the limited number of randomized studies and their methodological variability reduce confidence in effect estimates [11,13].

Second, the optimal program duration and timing are unclear. Aerobic capacity improvements typically require several weeks of training, yet many cardiac patients undergo surgery shortly after referral. Trials have used durations ranging from five days of IMT to several weeks of multimodal conditioning, making it difficult to determine the minimum effective dose [11,35].

Third, nutrition as a component of cardiac prehabilitation remains underexplored. Malnutrition, sarcopenia, and micronutrient deficiency are frequent in cardiac surgical patients, but few trials have tested structured nutritional intervention before surgery [44]. Recommendations are often extrapolated from general surgical guidelines rather than cardiac-specific evidence.

Fourth, there is a lack of standardization in exercise prescription. Studies often describe exercise intensity using nonspecific terminology (e.g., “moderate effort”), and IMT protocols vary markedly in workload and frequency [11,35]. This heterogeneity limits reproducibility and hampers comparison across trials.

Fifth, outcome endpoints lack uniformity. Current studies report diverse primary outcomes, including VO_2_max, pulmonary complications, length of stay, grip strength, or quality of life, complicating interpretation and meta-analysis [11,13]. Consistent, validated outcome sets are needed to benchmark progress across centers.

Finally, economic evaluation is largely absent from the cardiac literature. Although colorectal and hepatobiliary surgery prehabilitation demonstrates favorable cost-effectiveness, similar analyses have not been rigorously performed in cardiovascular cohorts [95]. Without financial evidence, widespread integration into routine care may face institutional resistance.

Future directions

Future development of cardiac prehabilitation will likely focus on expanding clinical infrastructure, refining patient selection, and incorporating digital technologies to maximize accessibility and benefit. Increasing alignment with Enhanced Recovery After Surgery (ERAS) cardiac pathways is expected, as these frameworks already emphasize multimodal preoperative optimization, early mobilization, and patient engagement [71]. The ERAS cardiac guidelines include key preoperative optimization measures such as smoking cessation (≥4 weeks), glycemic control, anemia management, and medication optimization that align closely with core components of prehabilitation. However, while ERAS provides a standardized perioperative care framework focused primarily on risk mitigation and protocolized optimization, prehabilitation extends this approach by incorporating proactive, multimodal interventions aimed at enhancing physiological reserve, including structured exercise training, nutritional conditioning, psychological support, and individualized risk modification [71]. Embedding the components of prehabilitation into ERAS protocols may standardize delivery, improve quality control, and accelerate adoption across cardiovascular centers.

Digital innovation will also shape the next phase of prehabilitation. Telemedicine and app-based remote monitoring platforms are gaining traction due to their ability to overcome travel distance, time constraints, and socioeconomic barriers. Telehealth-delivered cardiac rehabilitation has demonstrated effective patient engagement and capacity to improve outcomes, suggesting comparable feasibility for prehabilitation models [91]. Wearable technology and mobile applications may enable real-time exercise supervision, adherence prompts, and symptom tracking, providing continuous feedback loops between patients and clinical teams.

Individualized, strength-based exercise prescription is another emerging direction. Current protocols often adopt uniform low-to-moderate intensity programs; however, resistance and high-intensity interval methods may provide superior improvements in muscle mass and cardiopulmonary fitness, especially in frail or sarcopenic patients [37]. Tailoring intensity to baseline physiology may optimize training efficiency within short surgical waiting periods.

Biomarker-driven and machine-learning approaches to risk stratification may refine patient selection. Biomarkers such as NT-proBNP, iron indices, and inflammatory markers are increasingly used in cardiac risk assessment and may help identify patients most likely to benefit from prehabilitation [74]. Artificial intelligence modeling could integrate frailty indices, imaging data, comorbidities, and psychometric factors to prioritize high-risk patients who need early referral [98].

Finally, future research must involve larger multicenter randomized controlled trials designed with long-term clinical endpoints. Most current studies focus on short-term functional outcomes; survival, readmission, quality-adjusted life-years, and cost analyses are rarely evaluated. Lessons from colorectal and thoracic surgery show that sustained research investment can produce definitive evidence supporting routine prehabilitation [95]. Incorporating diverse populations, longer follow-up periods, and head-to-head comparisons against standard cardiac rehabilitation will be essential to determine the true impact and shape international guidelines.

## Conclusions

Current evidence supports the feasibility and safety of prehabilitation in adult cardiovascular surgery, with consistent improvements in preoperative functional capacity and signals toward reductions in postoperative pulmonary complications and hospital length of stay. Exercise-based interventions, particularly when combined with IMT, show the most robust benefits, while nutritional, psychological, and comorbidity-focused strategies remain less well studied in cardiac-specific populations. However, the evidence base is limited by heterogeneity in program design, small sample sizes, short intervention durations, and a lack of adequately powered randomized trials assessing clinically meaningful endpoints.

Important uncertainties persist regarding optimal patient selection, timing and intensity of interventions, and integration into existing perioperative care pathways such as ERAS. Future research should prioritize standardized, multimodal protocols, robust outcome measures, and scalable delivery models, including tele-prehabilitation, to support broader implementation. Addressing these gaps will be essential to determine the true clinical and system-level impact of prehabilitation in contemporary cardiovascular surgical care.
